# Evaluating blood levels of neuron specific enolase, chromogranin A, and circulating tumor cells as Merkel cell carcinoma biomarkers

**DOI:** 10.18632/oncotarget.4500

**Published:** 2015-07-02

**Authors:** Maria Rita Gaiser, Kenneth Daily, Jochen Hoffmann, Maik Brune, Alexander Enk, Isaac Brownell

**Affiliations:** ^1^ Department of Dermatology, University of Heidelberg, Heidelberg, Germany; ^2^ Dermatology Branch, Center for Cancer Research, National Cancer Institute, National Institutes of Health, Bethesda, MD, USA; ^3^ Department of Internal Medicine I and Clinical Chemistry, University of Heidelberg, Heidelberg, Germany

**Keywords:** merkel cell carcinoma, neuron specific enolase, chromogranin A, circulating tumor cells, EpCAM

## Abstract

**Background:**

Merkel cell carcinoma (MCC) is a rare, aggressive neuroendocrine skin cancer. Although used to monitor MCC patients, the clinical utility of neuron-specific enolase (NSE) and chromogranin A (ChrA) blood levels is untested. EpCAM-positive circulating tumor cells (CTC) reflect disease status in several epithelial tumors. Here we investigate the use of NSE and ChrA blood levels and CTC counts as biomarkers for MCC disease behavior.

**Methods:**

NSE and ChrA blood levels from 60 patients with MCC were retrospectively analyzed; 30 patients were additionally screened for CTC. Biomarker values were correlated to clinical parameters.

**Results:**

Despite routine use by some physicians, NSE and ChrA blood levels did not correlate with progression free survival, disease specific survival, or MCC recurrence. We found CTC in 97% of tested MCC patients. CTC counts were elevated in patients with active disease, suggesting their potential use in monitoring MCC.

**Conclusion:**

NSE and ChrA levels were not effective in predicting outcomes or detecting recurrences of MCC. In contrast, CTC counts have potential utility as a biomarker for MCC disease behavior.

## INTRODUCTION

Merkel cell carcinoma (MCC) is an aggressive skin cancer, and ∼80% of MCC tumors have DNA from the Merkel cell polyomavirus (MCV) integrated into their genome [1, 2]. Up to 80% of patients with MCC develop metastases [3]. The relative 5-year survival has been reported to be 64% for patients in stages I or II, 39% in stage III, and 18% in stage IV [4]. Aside from tumor stage, there are no robust prognostic biomarkers for MCC.

Biomarkers for disease prognosis and early detection of recurrences improve the care of cancer patients. Although a variety of biomarkers exist for malignancies such as breast [5] or colorectal cancer [6], there is an essential need for MCC biomarkers. Based on reports of elevated serum levels in patients with non-cutaneous neuroendocrine tumors [7–12], some institutions follow neuron specific enolase (NSE) and chromogranin A (ChrA) blood levels in MCC patients. However, this practice is not part of consensus management guidelines [13, 14] and the utility of NSE and ChrA as MCC biomarkers has not been tested.

Circulating tumor cells (CTC) can be detected in the bloodstream and hold potential as cancer biomarkers [15]. Recent studies have highlighted the prognostic significance of CTC [16, 17]. Most CTC-identifying assays use antibodies against epithelial markers, e.g. EpCAM [18, 19], the epithelial cell adhesion molecule that is known to be expressed on many carcinomas [20] including MCC [21].

To assess their utility as biomarkers for MCC, we have conducted a retrospective analysis of clinical tests used at our institution. Despite their routine use in this patient population, we found NSE and ChrA ineffective as prognostic markers or for detection of MCC recurrence. In contrast, our recent experience with measuring CTC in MCC patients suggests they could be developed as a useful biomarker for this aggressive cancer.

## RESULTS

### Patient characteristics

A total of 60 MCC patients were included in the study. Patient characteristics are shown in Table 1. Median follow up time was 43 months (range 3–182 months). At the last date of contact, 37 patients were alive with no evidence of disease, 6 were alive with disease, 9 had died of disease, and 8 had died of other causes. The estimated 5-year progression free survival (PFS) was 58.6% and 5-year disease specific survival (DSS) was 81.3%. Survival varied significantly with tumor stage for both PFS (*p*<0.05) and DSS (*p*<0.005) (Figure S1).

**Table 1:** Characteristics of patients included in the study and their Merkel cell carcinoma tumors

	Number	%
All patients	60	100
Male	37	62
Female	23	38
Median age at diagnosis: 70 y (range 33–90)		
Median follow up: 43 months (range 3–182)		
Patients with progression (60 total events)	24	40
		
Stage at diagnosis		
IA	20	33
IB	13	22
II	11	18
III	16	27
IV	0	0
		
Cases with FFPE tumor for immunostaining	46	77
NSE expression positive	46	100
CK20 expression positive	44	96
CD56 expression positive	42	91
ChrA expression positive	41	89
EpCAM expression positive	33	72
Merkel cell polyomavirus positive	30	65

### Tumor characteristics

We used immunostaining to assess potential biomarker expression in MCC tumors. Tumor tissue was available for 46 patients (77%). Of the tested samples, 100% stained positive for NSE, 96% for CK20, 91% for CD56, 89% for ChrA, 72% for EpCAM, and 65% for MCV (Table 1), confirming frequent expression of these MCC tumor markers. Among the immunostained cases, 67% were positive for both EpCAM and CD56, and 72% for EpCAM and CK20 suggesting frequent co-expression of these marker combinations in MCC tumors. Staining intensities for individual markers were graded from 0–2. For each immunohistochemical marker, staining intensity failed to correlate with PFS or DSS (Figure S2), suggesting they are not useful as prognostic markers.

### NSE and ChrA blood levels are not effective biomarkers

**Figure 1:**
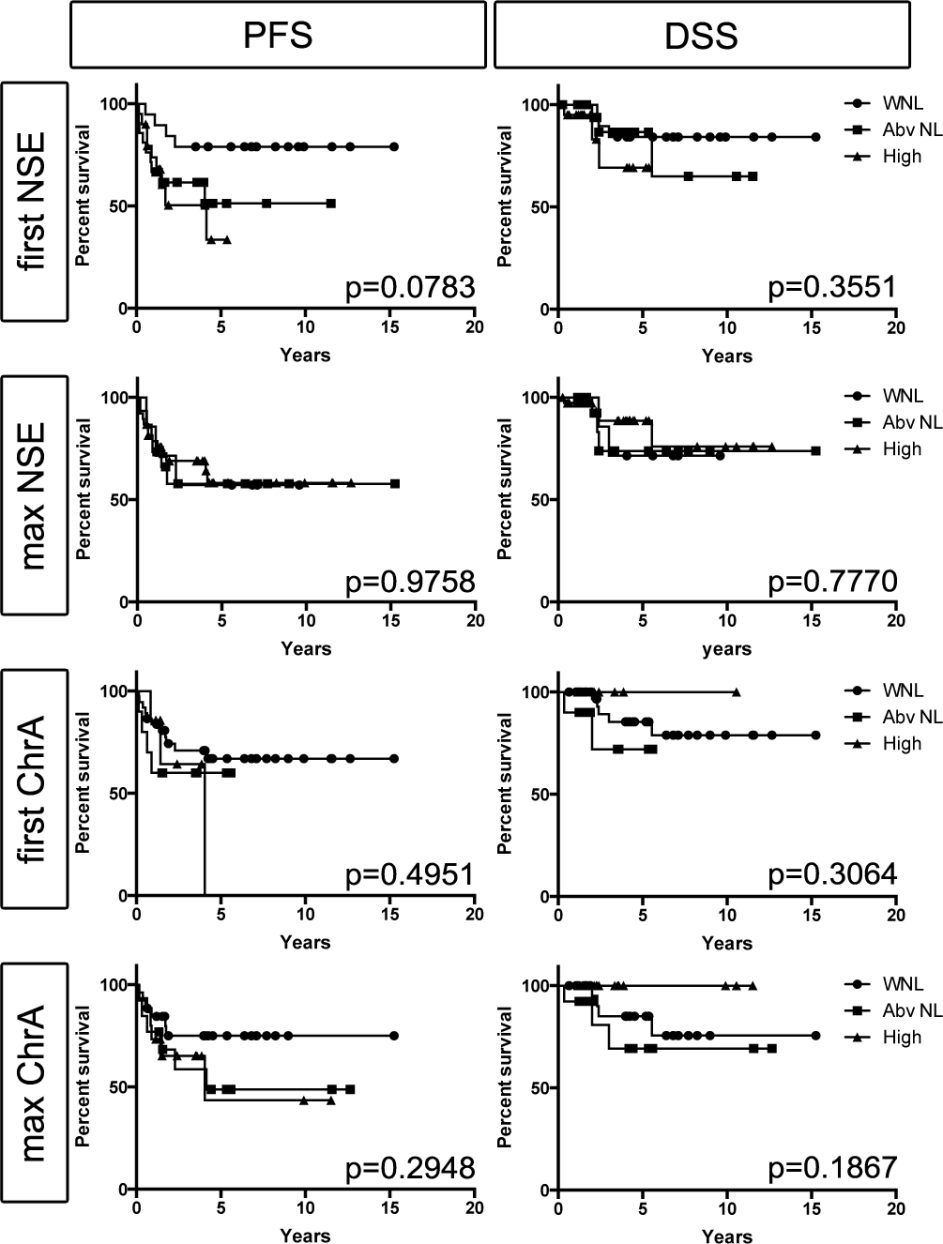
Blood levels of NSE or ChrA fail to correlate with PFS or DSS Kaplan-Meier survival estimates for first (initial assessment after diagnosis of MCC) or max (maximal level measured during follow up) NSE and ChrA values categorized as: within normal limits (WNL; circles), above normal (Abv NL; squares), or high (triangles) (*p*-values as indicated).

**Figure 2:**
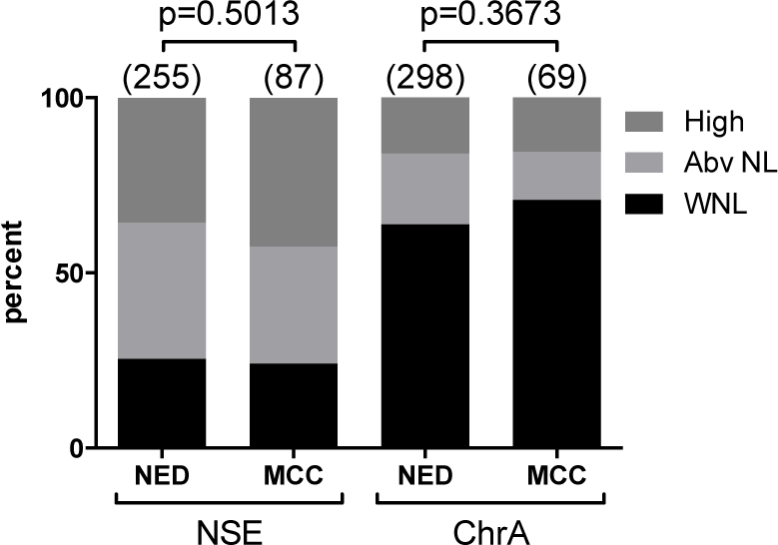
Blood levels of NSE or ChrA fail to correlate with tumor burden Distribution of all analyzed NSE and ChrA blood levels as percentages (total numbers as indicated) categorized as within normal limit (WNL; black), above normal (Abv NL; light grey), or high (dark grey) in patients with no evidence of disease (NED) compared to patients with tumor (MCC; 28 days prior to 56 days after tumor being present) (*p*-values as indicated).

Among the 60 study patients there was a total of 342 NSE and 367 ChrA blood level assessments. We analyzed NSE and ChrA levels as categorical variables, scored as either within normal limits (WNL), above normal (Abv NL), or high. There was no significant difference in PFS or DSS detected based on the patients' initial NSE or ChrA blood levels (first assessment after diagnosis of MCC, Figure 1), suggesting that initial NSE and ChrA levels are not effective as prognostic biomarkers. However, there was a trend (*p*=0.0783) toward PFS correlating with first NSE levels. Similar analysis of maximum NSE and ChrA levels failed to detect any difference in PFS or DSS, suggesting that elevations in these markers fail to correlate with disease progression (Figure 1). To directly assess if NSE or ChrA levels varied with tumor burden, we used data visualization of all lab values for each patient plotted with all treatment completion and disease recurrence events. There was no obvious trend associating tumor treatment or recurrence events to changes in NSE or ChrA levels (Figure S3). Finally we assessed the distribution of NSE and ChrA values drawn when patients had no evidence of disease (NED) versus patients with active or recent tumor burden (28 days prior to 56 days after tumor being present). There was no increased likelihood of finding elevated NSE or ChrA levels in patients with tumor versus those with NED (Figure 2). Taken together these results suggest that NSE and ChrA blood levels are not effective at predicting outcomes, following treatment response, or detecting recurrences in patients with MCC.

### Circulating MCC cells are readily detected and reflect tumor burden

We used the maintrac system to quantify CTC in a total of 30 MCC patients (Table 2), as well as 10 healthy control individuals (5 male, 5 female). All 30 patients were analyzed at least once, and 13 patients had 2 or more serial tests for a total of 56 assays. Controls were tested once. EpCAM+ circulating cells were detected in 90% of all MCC patient samples (median 450 cells/ml, range 0–11,000) and 60% of controls (median 175 cells/ml, range 0–1,000). Among the 30 patients, 29 (97%) had detectable CTC in at least one blood sample. Counts of EpCAM+ circulating cells in MCC patients were significantly higher than in controls (*p*<0.05). The detection of circulating EpCAM+ cells in healthy volunteers using maintrac suggests that a portion of the CTC counted in MCC samples are non-malignant cells.

**Table 2:** Characteristics of Merkel cell carcinoma patients monitored for CTC

	Number	%
All patients	30	100
Male	20	33
Female	10	67
Median age at diagnosis: 68 y (range 54–90)		
		
Stage at time of CTC monitoring		
IA	9	30
IB	6	20
II	2	7
III	9	30
IV	4	13
		
Individuals with circulating EpCAM+ cells/ml		
Patients (median: **450**, range 0–11,000)	29	97
Controls (*n* = 10) (median: **175**, range 0–1,000)*	6	60
		
Individuals with circulating EpCAM+ CD56+ cells/ml		
Patients (median: **150**, range 0–8,230)	23	77
Controls (*n* = 10) (median: **0**, range 0–0)^***^	0	0
		
Individuals with circulating EpCAM+ CK20+ cells/ml		
Patients (median: **220**, range 0–8,980)	24	80
Controls (*n* = 10) (median: **0**, range 0–630)*	3	30

* *p*<0.05

*** *p*<0.0005

**Figure 3:**
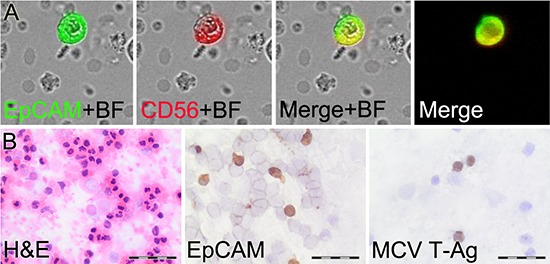
Detection of MCC circulating tumor cells (CTC) **A.** Laser Scanning Cytometry image of an EpCAM+, CD56+ CTC using immunofluorescence and brightfield (BF) detection. **B.** Light microscopy of peripheral blood cell suspensions stained with hematoxylin and eosin or immunoperoxidase staining of CTC with EpCAM and MCV large T-antigen (T-Ag). Scale bars=40 μm.

To more specifically detect CTC in MCC patients we added a second tumor marker to the assay (Figure 3A). In patient samples, the median percentage of CD56+ cells among EpCAM+ cells was 38% (range 0–100), and median percentage of CK20+ cells among EpCAM+ cells was 47% (range 0–100). EpCAM+, CD56+ CTC were detected in 23 of the 30 MCC patients (77%), whereas no (0%) control samples had EpCAM+, CD56+ double positive cells (*p*<0.0005). EpCAM+, CK20+ cells were detected in 24 MCC patients (80%) and 3 (30%) healthy controls (*p*<0.01). We also used immunoperoxidase staining for MCV large T-antigen on cell suspensions of two selected patients to confirm that MCV+ CTC are detectable in patient blood (Figure 3B). Unfortunately, we were unable to adapt fluorochrome labeled MCV large T-antigen antibody for routine use in our assay due to nonspecific staining. These results demonstrate that adding a second tumor marker can increase specificity of CTC analysis with EpCAM+, CD56+ double detection showing no false positives in healthy control patients.

**Figure 4:**
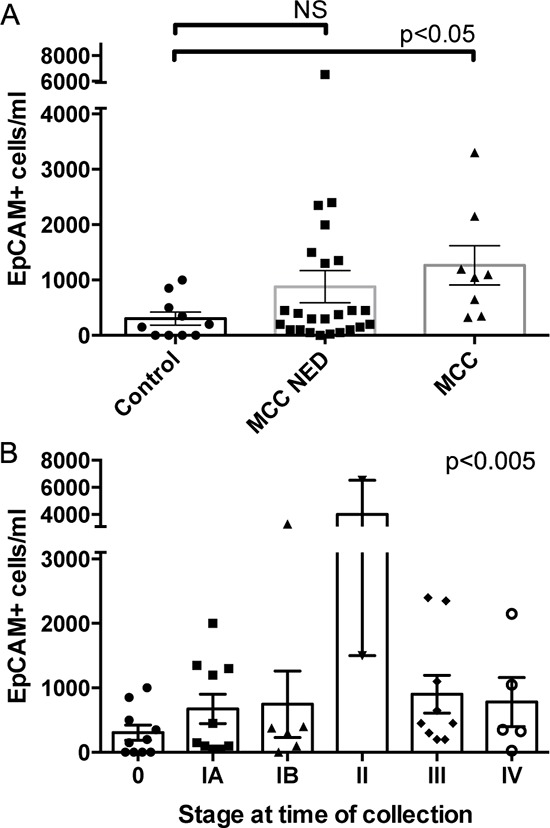
CTC counts correlate with MCC disease burden Absolute numbers and mean values of EpCAM+ cells/ml blood detected in **A.** healthy controls, MCC patients with no evidence of disease (MCC NED), and MCC patients with active or recent tumor (MCC); or **B.** patients with different disease stages at time of CTC assessment compared by one-way ANOVA (error bars=SEM, *p*-values as indicated).

There was insufficient follow up time after measuring CTC counts to assess their prognostic value. We used disease stage at time of blood collection (Table 2, control samples as stage 0) as a proxy for progression risk. The mean counts of EpCAM+ CTC were significantly different by disease stage (one-way ANOVA *p*<0.005), however a linear trend test was not significant reflecting a lack of correlation between increasing CTC counts and higher disease stage (Figure 4B). Similar results were seen with EpCAM+, CD56+ and EpCAM+, CK20+ CTC (Figure S4). One confounding issue in this analysis is that not all patient samples were drawn at the same point relative to their diagnosis, recurrence events, and tumor treatments (median time after diagnosis=38 months, range=0–148 months). The prognostic implications of MCC stage after disease progression are not well defined, and thus stage in this context may not portend eventual outcomes. Further follow up is needed before we can assess if CTC counts will correlate with disease progression.

To assess if CTC counts reflect MCC disease burden, we compared controls to NED patients and to patients with active disease (measurable tumor or treatment within the last 56 days). Of the 8 patients with active disease all 8 (100%) had detectable CTC regardless of the staining markers used. Moreover, despite the small sample size, patients with active disease had significantly higher CTC counts than controls for all staining combinations (Figure 4A and S4, *p*<0.05), suggesting that CTC counts may be useful in following the extent of disease or detecting recurrences in patients with MCC. Many MCC patients with NED had CTC counts comparable to those seen in healthy controls, but some had elevated CTC counts. It is unclear if the NED patients with elevated CTC counts had occult disease or are at higher risk for disease progression.

**Figure 5:**
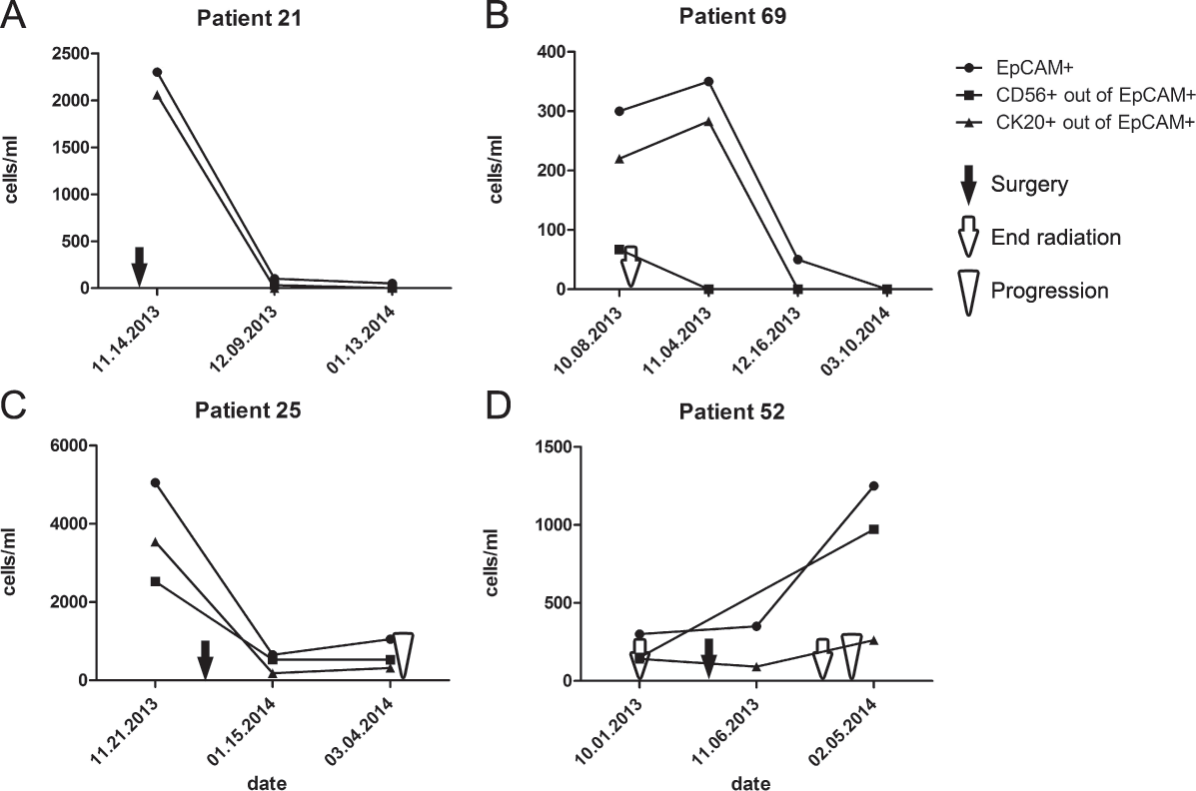
Longitudinal changes in CTC counts reflect MCC treatment response and tumor progression in individual patients **A.** Patient 21 had surgery of a local recurrence on the 11th of November 2013 (black arrow), CTC numbers decreased within 4 weeks and then stayed stable during the next 4 weeks. **B.** Patient 69 had radiotherapy from the 4th of September 2013 until the 9th of October (white arrow), followed by a therapy with interferon α2b over the next 18 months, CTC numbers increased during the first weeks of interferon treatment, but then decreased dramatically. **C.** Patient 25 had surgery of regional metastases on the 27th of December 2013 (black arrow), CTC numbers decreased within 4 weeks followed by a CTC increase and the diagnosis of distant metastases on the 5th of March 2014 (white arrowhead). **D.** Patient 52 completed radiotherapy on the 1st of October 2013 (white arrow); on the 23rd of October a regional LN metastasis was resected (black arrow) and treated with radiotherapy. Shortly after completing radiation (white arrow), a distant metastasis was diagnosed on the 30th of January 2014 (white arrowhead).

In patients with multiple CTC observations, we were able to examine CTC counts over time. Patients without any disease related events showed consistent CTC numbers over time with deviations of no more than 20%. Four patients were monitored while undergoing tumor treatment, and all displayed decreasing CTC numbers (Figure 5A–5C). Only 2 patients developed new metastases during serial monitoring, both associated with increasing CTC numbers (Figure 5C, 5D). Although anecdotal, these observations further suggest that CTC counts reflect MCC disease status and could be used to effectively monitor for treatment responses and detect recurrences.

## DISCUSSION

We found that NSE and ChrA levels are not highly effective biomarkers to predict progression or detect recurrent MCC. In contrast, CTC counts demonstrate potential utility as a biomarker for MCC disease behavior.

Elevated blood levels of NSE and ChrA have been reported in MCC patients [22–27]. However, their diagnostic and prognostic value has only been investigated in single cases or small patient groups. A comparison between ChrA and NSE levels in neuroendocrine tumor (NET) patients demonstrated elevated ChrA in 25% and NSE in 50% of four MCC patients [28]. In contrast, Cimitan et al. found normal ChrA levels in six MCC patients [29]. Our observations from 60 patients over 259 person-years suggest changes in NSE and ChrA are not clinically meaningful in following patients with MCC. Most MCC tumors express NSE and ChrA, however both markers are also expressed in normal tissues [11, 30], requiring sufficient tumor burden to alter blood levels [7]. Thus, it is not surprising to find that NSE and ChrA might be more specific as biomarkers for lower grade NETs and less effective in aggressive MCC. However, as its retrospective design and inconsistent testing across the patient population limit our study of NSE and ChrA in MCC, it is possible that a weak correlation between these markers and MCC went undetected. A regimented prospective analysis would be required to investigate this possibility.

CTC enumeration has proven useful in establishing prognosis for patients with breast, colon, liver, and prostate cancer [16–19]. Breast CTC can reflect response to chemotherapy, predict relapse, and serve as markers for tumor aggressiveness [19]. Interestingly, breast cancer CTC can be found up to 22 years after mastectomy in NED patients [31]. Similarly, we detected elevated CTC counts (480 EpCAM+, CD56+ cells/ml) in a NED patient 11 years after MCC diagnosis. The source and nature of CTC detected in long-term cancer survivors remains unclear, and raises questions about their tumorigenic potential.

Numerous assays can detect CTC at single-cell resolution in peripheral blood [32], however some methods have shortcomings to consider. Flow cytometry cannot differentiate morphologically characteristic tumor cells from nonspecific events [33]. Density gradient purification leads to loss of relevant cells [34]. To date, CTC in the blood of MCC patients have been described in a handful of case reports and one case series [35–38]. Blom et al. [38] analyzed CTC from 34 MCC patients using the CellSearch system (Veridex LLC) that is based on magnetic bead enrichment. In contrast to maintrac, CellSearch is dependent on cells expressing a minimum level of surface antigen to be retained in the magnetic field, uses a large blood volume (7.5ml), and may result in cell destruction [39–42]. As a result, CellSearch shows lower sensitivity than other methods. Our study used the highly-sensitive maintrac assay that directly stains and quantifies cells from a single milliliter of patient blood.

Using the maintrac system we detected EpCAM+ CTC in 97% of MCC patients with a median of 450 cells/ml, and EpCAM+ CD56+ CTC in 70% of MCC patients with a median of 150 cells/ml. This contrasts with Blom et al. [38] who detected EpCAM+, CK8+ CTC in 41% of patients with a median of only 2 cells/7.5ml. Both studies demonstrated an association between CTC detection and extent of disease, as well as CTC reductions in response to therapy. In contrast to their findings, we found EpCAM+, CD56+ CTC in 13 of 22 (59%) NED patients, whereas Blom et al. did not detect CTC in NED patients. At the same time, their study failed to detect CTC in 15 of 27 cases (56%) that had active disease, whereas we found CTC in 8 of 8 samples (100%) from patients with active disease. Their study demonstrated a correlation between disease outcomes and CTC detection. Longer follow up is needed to determine the prognostic value of CTC levels in our study population.

Using maintrac we detected circulating EpCAM+ cells in some healthy controls. EpCAM is often expressed on epithelial tumors but also on some normal epithelia [20, 43], and normal epithelial cells can circulate in blood under certain circumstances [44, 45]. To increase the specificity of CTC detection, we used anti-EpCAM in combination with anti-CK20 [46, 47] or anti-CD56 [48–51]. Blood cells lack EpCAM expression, allowing for the exclusion of CK20 expression in granulocytes [52], and CD56 on NK T-cells [53]. However, EpCAM+, CK20+ cells in healthy volunteers suggest that some circulating cells may be normal intestinal epithelium that co-expresses both markers [54, 55]. In contrast, no circulating EpCAM+, CD56+ cells were found in healthy volunteers, making it the most specific marker combination tested.

## CONCLUSIONS

We found that despite the reported utility of NSE and ChrA blood levels as biomarkers for low grade NETs, clinical use of NSE and ChrA levels in MCC patients failed to correlate with outcomes, disease progression, or tumor burden. In the same patient population we found that maintrac detection readily identified EpCAM+ CTC with high sensitivity. Moreover, adding CD56 as a second tumor marker increased the specificity of CTC detection. Although CTC counts reflected tumor burden, additional follow up is needed to determine how CTC correlate with disease outcomes. The use of CTC as biomarkers for MCC will require further development and validation, but our results and those of Blom et al. suggest CTC may be useful in the staging and longitudinal monitoring of MCC.

## MATERIALS AND METHODS

### Patients

Patients treated at the University of Heidelberg for MCC between 1998 and 2014 with at least one laboratory evaluation of blood NSE or ChrA levels were included in the study. MCC diagnosis was verified histopathologically. Clinical and biographical data were abstracted in a systematic chart review completed in October 2014. MCC staging was classified according to 7th edition AJCC guidelines [4].

All procedures have approved by the University of Heidelberg medical ethics committee (approval number S570/2013). Informed consent has been obtained.

### Analysis of NSE and chrA blood levels

Blood samples were analyzed for NSE and ChrA in the Heidelberg University Hospital's central laboratory. NSE (ug/ml) in serum was categorically interpreted as WNL <17, above normal ≥17–24.3, or high ≥24.3. Until December 2012 ChrA (U/ml) was measured in plasma, and after December 2012 ChrA (ng/ml) was measured in serum. A period of parallel analyses in plasma and serum assured good correlation between the two systems. A common categorical scale for ChrA levels was used as follows. ChrA plasma: WNL <25, above normal ≥25–53.75, high ≥53.75. ChrA serum: WNL <84.7, above normal ≥84.7–105.65, high ≥105.65.

### Detection of CTC

Patients treated for MCC between September 2013 and May 2014 who consented to participate were screened for CTC. Detection of CTC (cells/ml blood) using the maintrac system (SIMFO, Bayreuth, Germany) was performed as previously described [56]. EpCAM staining was used to identify all putative tumor cells, and CD56 or CK20 staining was added to confirm MCC tumor cells. Only appropriately stained cells that met the morphological criteria of a tumor cell were counted as CTC.

### Immunohistochemistry

Formalin-fixed, paraffin-embedded (FFPE) tissue was cut in 2 μm sections, stained using standard immunohistochemistry protocols, and visualized using the Envision System (Dako) as described by the manufacturer. For immunohistochemical staining of CTC, cell suspensions were dropped on slides, air-dried and stained with the primary antibodies.

### Statistics

Progression free survival (PFS) and disease specific survival (DSS) were calculated from the date of first treatment until the date of first progression, death, or last follow-up. Two-tailed *p*-values were used for all comparisons.

Detailed methods are available in the Supplementary Materials.

## SUPPLEMENTARY DATA


