# Supplementary material for: Evaluating blood levels of neuron specific enolase, chromogranin A, and circulating tumor cells as Merkel cell carcinoma biomarkers
Source: Oncotarget. 2015 Jul 2;6(28):26472–82. doi: 10.18632/oncotarget.4500 (PMC4694915; doi:10.18632/oncotarget.4500)
Supplement: Supplementary file 1 [file oncotarget-06-26472-s001.pdf]

SUPPLEMENTARY DATA

Analysis of NSE and ChrA blood levels

Plasma and serum samples were analyzed for NSE and ChrA in the central laboratory of the Heidelberg University Hospital applying validated and accredited analytical methods. Briefly, NSE (µg/ml) in serum was quantified on a Cobas e411 analyzer via a chemiluminescence immunoassay according to the manufacturer's instructions (Roche, Mannheim, Germany). The laboratory range for normal NSE was <17. We used the median of all patient values above the normal range as a cut off between "above normal" and "high" values. Thus NSE was categorically interpreted as within normal limits (WNL) <17, above normal (Abv NL) ≥17–24.3, or high ≥24.3. Until December 2012 ChrA (U/ml) was quantified in EDTA plasma applying a peroxidise-based colorimetric immunoassay (Dako Cytomation, Glostrup, Denmark) at 450 nm on an anthos 2010 microplate reader (anthos Mikrosysteme GmbH, Krefeld, Germany). After December 2012 ChrA (ng/ml) was measured in serum on a Kryptor compact PLUS (BRAHMS GmbH, Hennigsdorf, Germany) applying a time-resolved amplification of cryptate emission (TRACE)-based immunoassay (BRAHMS GmbH). A period of parallel analysis in plasma and serum samples assured good correlation between the old and new systems for ChrA measurement. To provide a common categorical scale, ChrA levels were interpreted as was done for NSE as follows. Range ChrA plasma: WNL <25, Abv NL ≥25–53.75, or high ≥ 53.75. Range ChrA serum: WNL <84.7, Abv NL ≥84.7–105.65, or high ≥105.65.

Detection of CTC

Patients treated for MCC between September 2013 and May 2014 who consented to participate were screened for CTC. Every blood sample for CTC analysis was subjected to a complete blood count to ensure cell numbers were within the normal physiological range. Detection of CTC (cells/ml blood) using the laser scanning cytometer-based maintrac system (SIMFO, Bayreuth, Germany) was performed as previously described [1]. Briefly, 1ml of EDTA blood was subjected to red blood cell lysis using 10ml of erythrocyte lysis solution (Qiagen, Hilden, Germany) for 10min in the cold. After centrifugation, the white cell pellet was diluted in 1ml of PBS and incubated with primary fluorochrome labeled antibodies against EpCAM (1:10, Miltenyi, clone HEA, FITC), and CD56 (1:5, Beckman Coulter, clone N901, PE), or CK20 (1:10, Santa Cruz, clone G20, PE). Immunoperoxidase staining for MCV large T-antigen (1:10, Santa Cruz, clone CM2B4) was detected by standard DAB detection. Cells stained for CK20 or MCV large T-antigen were permeabilized prior to incubation by adding detergent to the washing buffer. Cell suspensions were either added to a microtiter plate (Greiner Bio-One, Frickenhausen, Germany) to acquire a series of photos of at least 100 cells with subsequent manual counting, or placed on adhesion slides (Menzel, Braunschweig, Germany) for direct manual counting of 50–100 cells. If stained cells were infrequent (<10 cells per photo), at least 300 cells were counted. EpCAM staining was used as the first screening test to identify all putative tumor cells; anti-CD56 or anti-CK20 was added to confirm MCC tumor cells. Only appropriately stained cells that met the morphological criteria of a tumor cell were counted as CTC.

Immunohistochemistry

Formalin-fixed, paraffin-embedded (FFPE) tissue was cut in 2 µm sections, stained using standard immunohistochemistry protocols, and visualized using the Envision System (Dako) as described by the manufacturer. The following monoclonal antibodies were used: anti-EpCAM (1:100, Dako, clone berEP4), anti-CD56 (1:100, Leica, clone 1B6), anti-CK20 (1:200, Dako, clone Ks20.8), anti-NSE (1:200, Dako, clone BBS/NC/VI-H14), anti-ChrA (prediluted, Linaris, clone LK2H10), anti-MCV large T-Antigen (1:100, Santa Cruz clone CM2B4). Staining intensity of tumor sections was scored as 0=no staining, 1=intermediate staining, or 2=strong staining by a single blinded observer. For immunohistochemical staining of CTC, cell suspensions were dropped on slides, air-dried and stained with the primary antibodies in conditions as described above. For morphological controls, cell suspensions were stained with hematoxylin for 2 min.

Statistics

Progression free survival (PFS) and disease specific survival (DSS) were calculated from the date of first treatment until the date of first progression, death, or last follow-up. The probability of PFS or DSS as a function of time was estimated by the Kaplan-Meier method. The statistical significance of the difference among a set of Kaplan-Meier curves was determined by the Mantel-Cox log-rank test. Comparisons between two population means were made using an unpaired *t*-test with Welch correction. Comparisons among

multiple population means used one-way ANOVA. Nonparametric population comparisons were made using the Mann-Whitney test. Associations between categorical parameters were determined using the Cochran–Armitage test for trend. Two-tailed *p*-values were used for all comparisons.

REFERENCES

1. Pachmann K, Clement JH, Schneider CP, Willen B, Camara O, Pachmann U, Hoffken K. Standardized quantification of circulating peripheral tumor cells from lung and breast cancer. Clin Chem Lab Med. 2005; 43:617–627.

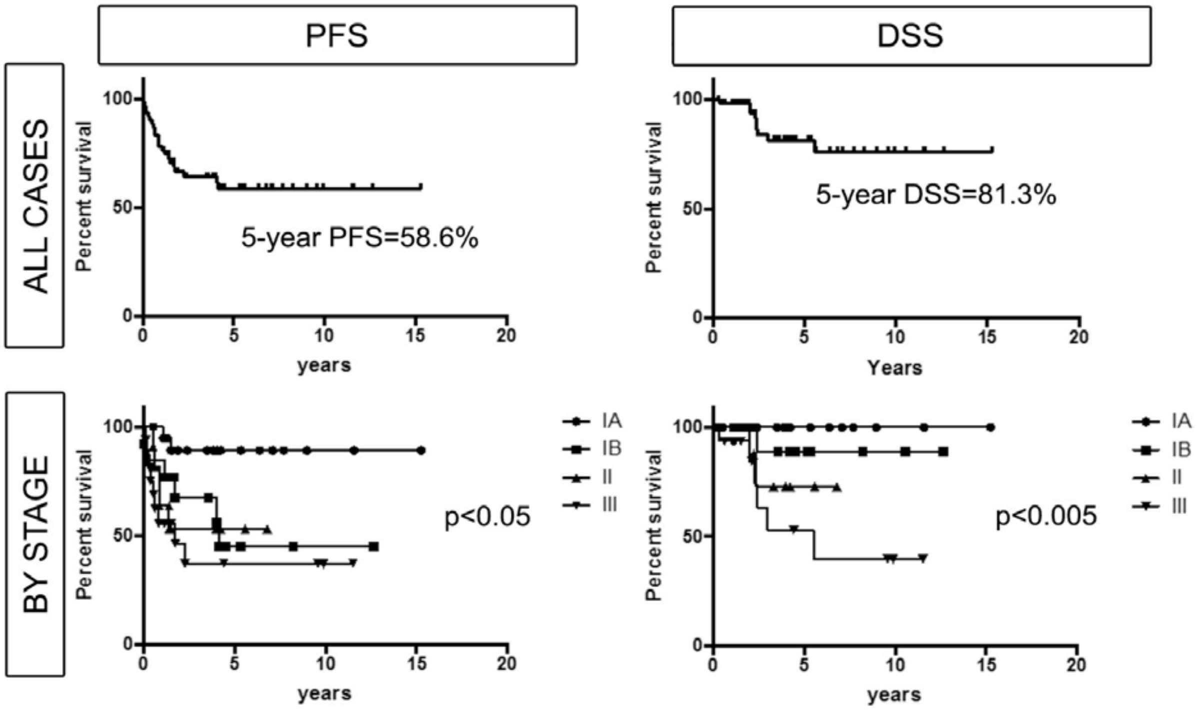

Supplementary Figure S1: MCC tumor stage correlates with PFS (left column) and DSS (right column). Kaplan-Meier survival curves. (5-year survival estimates and *p*-values as indicated).

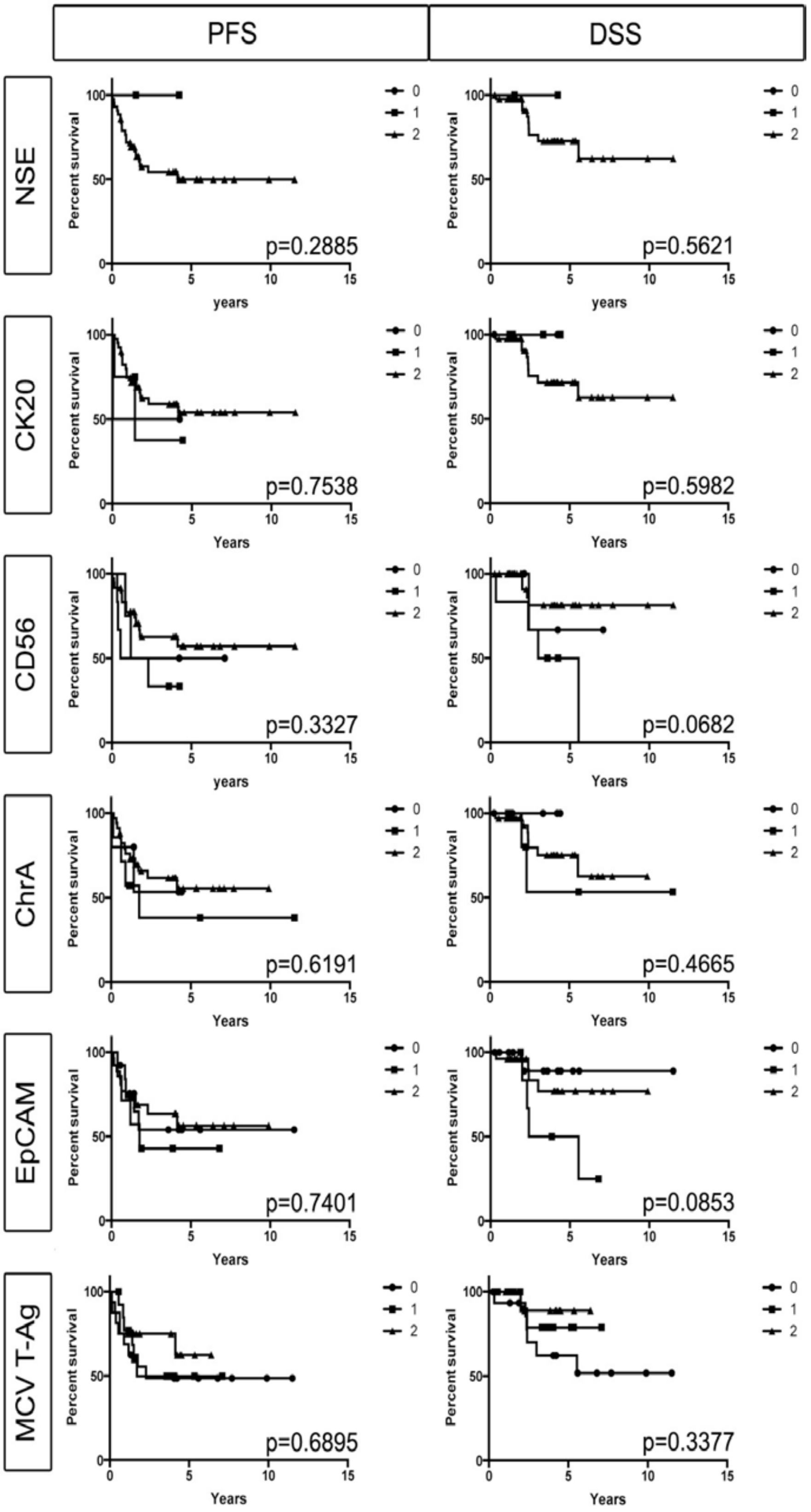

Supplementary Figure S2: Immunohistochemical MCC markers fail to predict PFS or DSS. Kaplan-Meier survival estimates fail to detect significant difference is PFS (left column) and DSS (right column) based on the staining intensities of MCC markers, staining intensities are graded as 0=no staining; 1=intermediate; 2=strong staining (*p*-values as indicated).

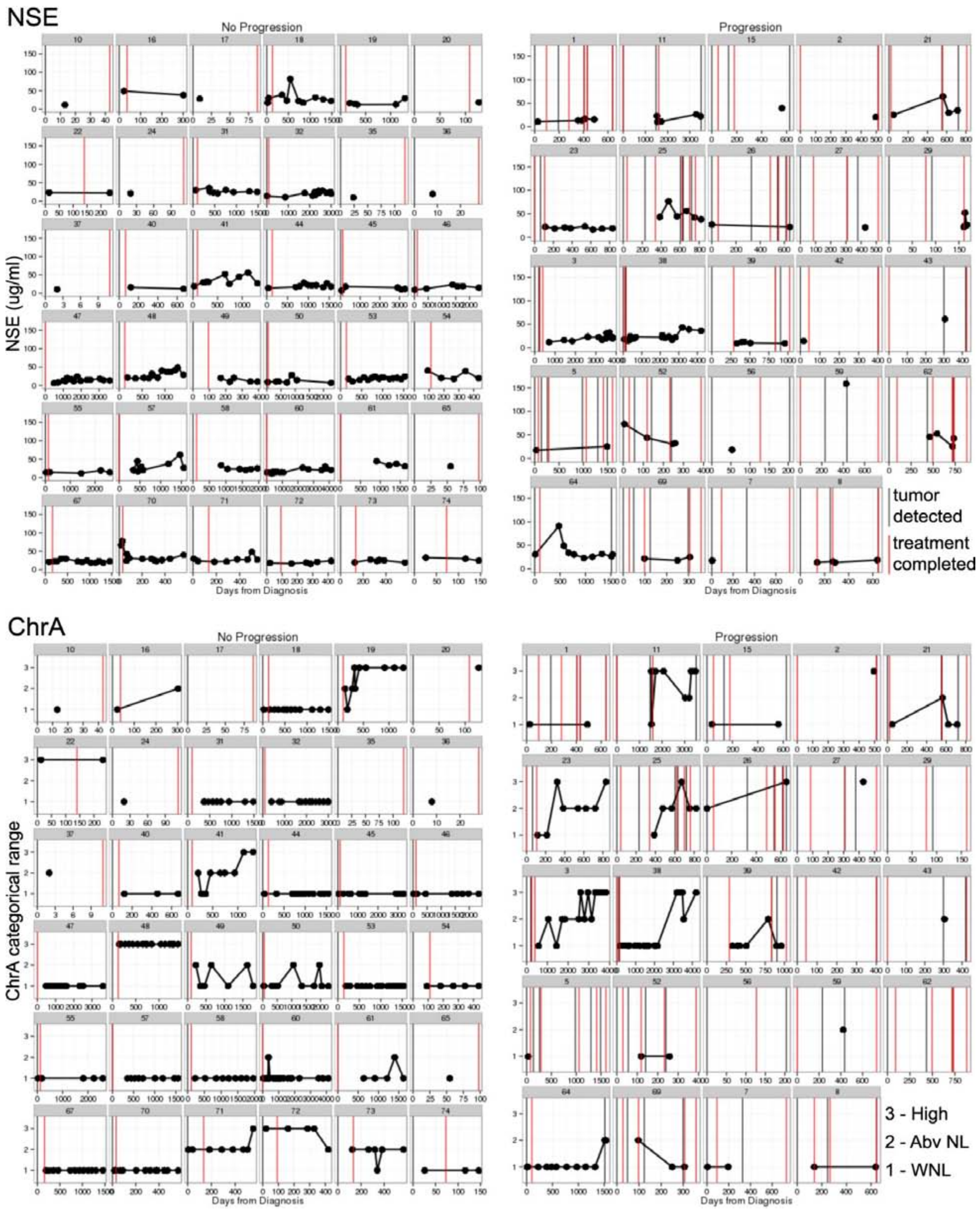

Supplementary Figure S3: Data visualization fails to find temporal associations between NSE or ChrA blood values and changes in MCC disease status. NSE (upper) and ChrA (lower) levels observed at time points after diagnosis of MCC, graphed with dates of new tumor detection (grey vertical lines) and tumor treatment completion (red vertical lines) for patients with no progression events (left) and patients with tumor progression events (right). X-axis, days from initial diagnosis. Y-axis, tumor marker levels in µg/ml (NSE) or categorical interpretation (ChrA): within normal limits (WNL, 1), above normal (Abv NL, 2), or high (3). See https://github.com/BrownellLab/MCC_NSE_CHA/ for the data and code for this analysis.

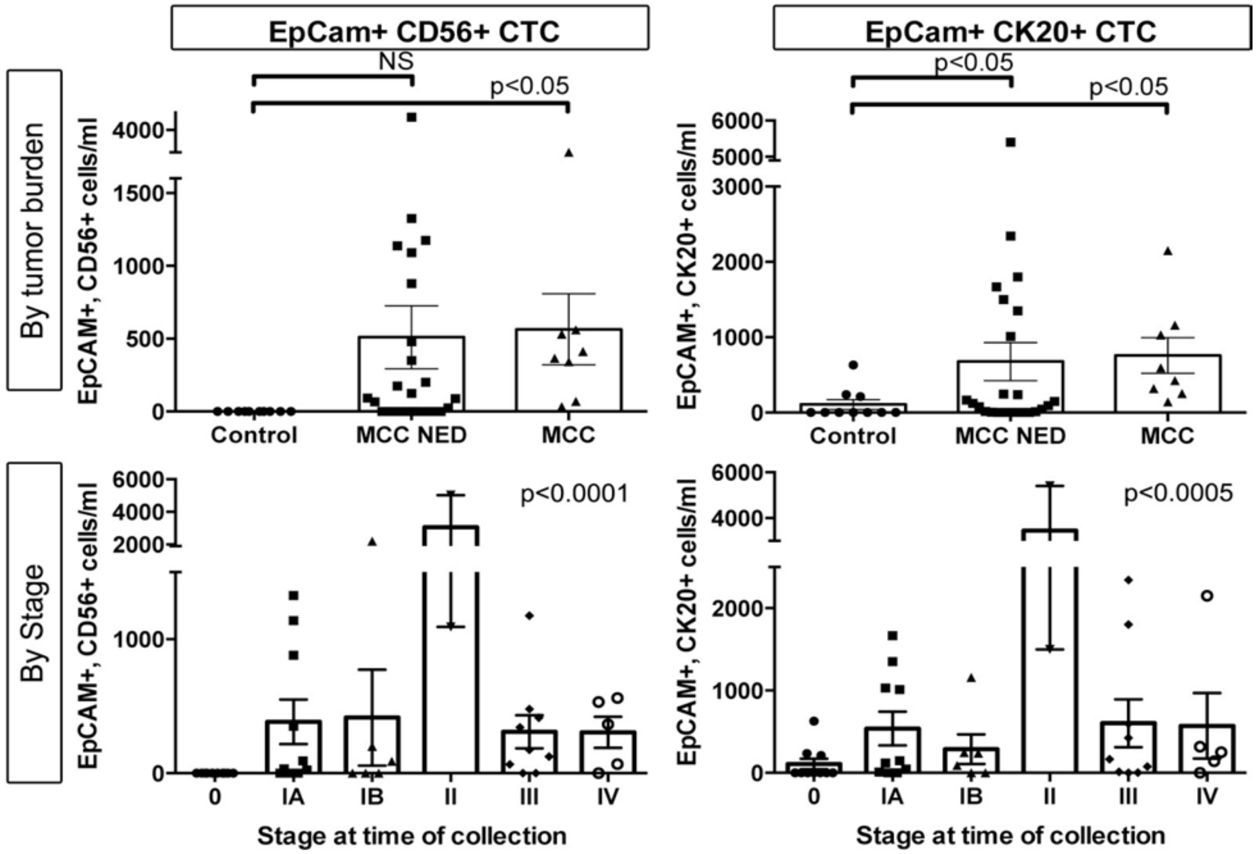

Supplementary Figure S4: CTC counts correlate with MCC disease burden. Absolute numbers and mean values of EpCAM+, CD56+ cells/ml (left column) and EpCAM+, CK20+ cells/ml (right column) detected in healthy controls, patients with no evidence of disease (MCC NED), and patients with tumor (MCC); or patients with different disease stages at time of CTC assessment (error bars=SEM, *p*-values as indicated).
